# Alkaloids from Fritillariae Cirrhosae Bulbus - progress on their pharmacological activities

**DOI:** 10.3389/fphar.2026.1826161

**Published:** 2026-05-20

**Authors:** Liu Xinyu, Qin Qianxi, Mahmoud Soliman, Du Cheng, Zhao Liangwenyao, Ehab Gamal, Shi Chen, Liu Zhiwei, Cheng Linli

**Affiliations:** 1 College of Veterinary Medicine, China Agricultural University, Beijing, China; 2 National Key Laboratory of Veterinary Public Health Safety, Beijing, China; 3 Science and Technology and Agriculture and Animal Husbandry Bureau of Zoigê County, Aba, Sichuan, China; 4 Sichuan Baihe Pasture Co., Ltd., Aba, Sichuan, China

**Keywords:** alkaloids, development of Chinese botanical drug, fritillariae cirrhosae bulbus, pharmacological activity, utilization of Chinese botanical drug

## Abstract

Fritillariae Cirrhosae Bulbus is a valuable and authentic medicinal material widely used in traditional Chinese medicine to treat respiratory diseases. Modern medical research shows that alkaloids, the main metabolites of Fritillariae Cirrhosae Bulbus, not only have expectorant and antitussive effects and can disperse swelling, but also have important clinical value and broad prospects for feed development in many areas, such as anti-tumor, antihypertensive, and antioxidant activities. This review systematically summarizes the types of alkaloids found in Fritillariae Cirrhosae Bulbus, including steroidal and isosteroidal forms, as well as six categories of pharmacological effects, such as antitussive, antiasthmatic, and antibacterial activities. It identifies current research challenges, namely, the unclear identification of active metabolites and the lack of elucidation of their mechanisms of action. This work offers a reference for further research and clinical application of Fritillariae Cirrhosae Bulbus.

## 1 Introduction

Fritillariae Cirrhosae Bulbus, the dried bulb of various perennial herbaceous plants belonging to the genus *Fritillaria*, is a quintessential, authentic medicinal material originating from the Sichuan region. The source plants of Fritillariae Cirrhosae Bulbus, according to the Chinese Pharmacopoeia (2020 edition), the botanical drug Fritillariae Cirrhosae Bulbus is derived from the following six *Fritillaria* species: *Fritillaria cirrhosa* D. Don, *F. unibracteata* P. K. Hsiao and K. C. Hsia, *F. przewalskii* Maxim., *F. delavayi* Franch., *F. taipaiensis* P. Y. Li, and *F. thunbergii* Miq. Regional differences exist in source species: *F. cirrhosa* and *F. unibracteata* are predominantly used in Sichuan and Chongqing; *F. przewalskii* is common in Qinghai and Gansu; and *F. delavayi* is mainly sourced from Tibet (Jiang et al., 2016; Guo et al., 2019). In Traditional Chinese Medicine (TCM) theory, based on morphological characteristics, Fritillariae Cirrhosae Bulbus is categorized into Songbei, Qingbei, Lubei, and cultivated varieties. It possesses a sweet and cold property and is attributed with effects that clear heat, resolve phlegm, relieve cough, alleviate dyspnea, dissipate nodules, and reduce swelling. Clinically, it is indicated for externally contracted coughs, deficiency-consumptive coughs, chest tightness due to fire stagnation, and conditions such as sores, swellings, and scrofula (Xin et al., 2022; Peng et al., 2022; Xie et al., 2022; Chen et al., 2019; Guo et al., 2019; Hou et al., 2023). Modern preclinical *in vivo* and *in vitro* studies have revealed that Fritillariae Cirrhosae Bulbus contains multiple chemical metabolites with anti-tumor, antihypertensive, antioxidant, anti-inflammatory, and antibacterial activities. These include alkaloids (Liu et al., 2020; Wang D. et al., 2016), saponins (Huang et al., 2021; Han et al., 2025), polysaccharides (Shiwei, 2022), volatile oils (Wang and Li, 2013), terpenoids (Bhat et al., 2022), and trace elements (Cui et al., 2021). Among these, alkaloids are recognized as the primary active metabolites (Lu et al., 2022), underscoring their significant medicinal value and promising research prospects.

Due to its growth in alpine and plateau regions above 2,000 m in altitude, with a distribution largely confined to the southwestern plateau climate zone (Peng et al., 2022), *Fritillaria* species exhibit prolonged seed dormancy and low natural germination rates. Compounded by the overexploitation of wild resources (Guo et al., 2019; Wang et al., 2017), the wild Fritillariae Cirrhosae Bulbus has become scarce and low-productive. Consequently, it is classified as a rare and endangered authentic medicinal material and is designated as a Grade III protected wild medicinal species in the “List of Wild Medicinal Materials under State Protection” (Xiao et al., 2025). Although some large-scale cultivation bases currently exist in China, the quality of cultivated products is often suboptimal. With the market demand far exceeding supply, the price of Fritillariae Cirrhosae Bulbus remains high (Cunningham et al., 2018). Therefore, its application in animal production has not been widespread. However, the herbal processing residues, which are referred to as by-products generated during medicinal processing, still possess significant medicinal value (Liu et al., 2019) and can be utilized as raw materials for veterinary pharmaceuticals and animal feed additives. This endows it with broad application prospects and market potential in areas such as disease prevention and treatment, production efficiency improvement, and product quality enhancement.

Alkaloids are a prominent focus in the study of the chemical metabolites of Fritillariae Cirrhosae Bulbus. They are present in the dried bulbs of Fritillariae Cirrhosae Bulbus. To date, their main extraction methods include percolation extraction, reflux extraction, enzymatic extraction, supercritical fluid extraction, and others. Many studies have demonstrated that alkaloids are important active metabolites in this medicinal material (Chen CC. et al., 2020). Consequently, determining the chemical structures of these alkaloids, exploring their potential pharmacological activities, and investigating their mechanisms of action are of great significance for the rational utilization of Fritillariae Cirrhosae Bulbus resources. This paper provides a review of the types and pharmacological effects of alkaloids found in Fritillariae Cirrhosae Bulbus, as well as their application prospects, aiming to offer a reference for the efficient application of Fritillariae Cirrhosae Bulbus in animal production.

## 2 Types of alkaloids in Fritillariae Cirrhosae Bulbus

**FIGURE 1:**
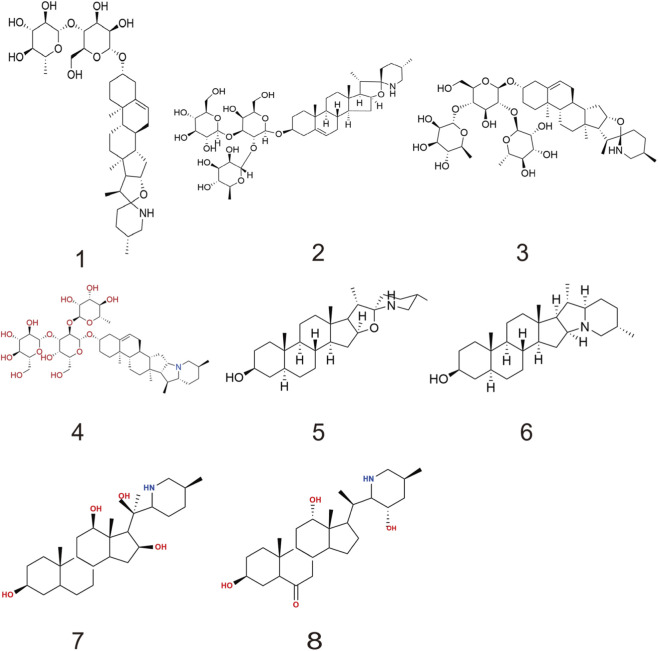
Chemical structure of steroidal alkaloids in fritillariae cirrhosae bulbus.

**FIGURE 2:**
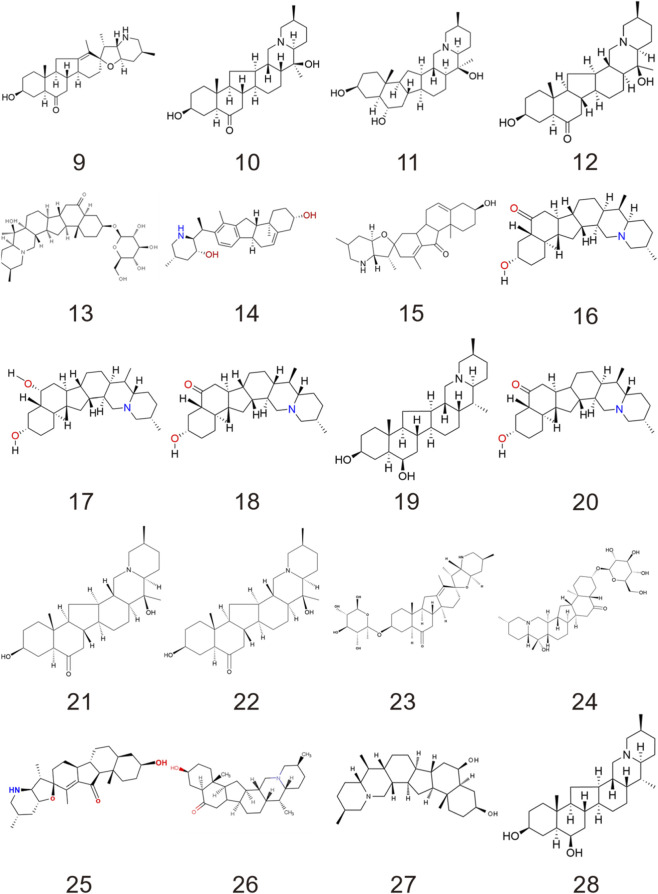
Chemical structure of isosteroidal alkaloids in fritillariae cirrhosae bulbus.

To date, hundreds of alkaloids have been isolated from medicinal plants of the genus *Fritillaria*. It is known that Fritillariae Cirrhosae Bulbus contains a variety of steroidal alkaloids, which can be classified into steroidal, isosteroidal, and other types based on their steroidal skeleton structures (Zhou et al., 2016). Among them, Isosteroidal alkaloids are a subclass of steroidal alkaloids characterized by a rearranged C-nor-D-homosteroid skeleton, typically derived from cholestane or spirostane precursors. They are further divided into cevanine, veratramine, jervine, and solanidine types based on ring system modifications (Lu et al., 2022; Zhou et al., 2010). Table 1 summarizes 30 representative compounds with well-defined pharmacological activities that have been reported from Fritillariae Cirrhosae Bulbus. The chemical structures of some alkaloids are shown in Figures 1, 2.

**TABLE 1:** Several alkaloids in Fritillariae Cirrhosae Bulbus.

Sort	Compound	Original botanical sources	Molecular formula	Number	References
Steroidal	Khasianine	Multiple botanical origins	C_39_H_63_NO_11_	1	Lu et al. (2022)
	Solasonine	Multiple botanical origins	C_45_H_73_NO_16_	2	
	Solamargine	Multiple botanical origins	C_45_H_73_NO_15_	3	
	Solanine	Multiple botanical origins	C_45_H_73_NO_15_	4	
	Tomatidine	Multiple botanical origins	C_27_H_45_NO_2_	5	
	Demissidine	Multiple botanical origins	C_27_H_45_NO	6	Wang et al. (2016b)
	Chuanbeinone A	*F. cirrhosa* or *F. unibracteata*	C_27_H_47_NO_4_	7	Chen et al., 2020b; Cao (2008)
	Chuanbeinone B	*F. cirrhosa* or *F. unibracteata*	C_27_H_45_NO_4_	8	
Isosteroidal	Peimisine	Multiple botanical origins	C_27_H_41_NO_3_	9	Chen et al. (2020a)
	Sipeimine	Multiple botanical origins	C_27_H_43_NO_3_	10	
	Peimine	Multiple botanical origins	C_27_H_45_NO_3_	11	Chen et al. (2020a), Chang et al. (2020)
	Peiminine	Multiple botanical origins	C_27_H_43_NO_3_	12	
	Edpetiline	Multiple botanical origins	C_33_H_53_NO_8_	13	Lu et al. (2022)
	Veratramine	Multiple botanical origins	C_27_H_39_NO_2_	14	
	Jervine	Multiple botanical origins	C_27_H_39_NO_3_	15	
	Chuanbeinone	*F. cirrhosa* or *F. unibracteata*	C_27_H_43_NO_2_	16	Wang et al. (2016b)
	Ebeiedine	*Fritillaria cirrhosa* or *Fritillaria hupehensis*	C_27_H_45_NO_2_	17	Wu et al. (2018)
	Ebeiedinone	*Fritillaria cirrhosa* or *Fritillaria hupehensis*	C_27_H_43_NO_2_	18	
	Delavine	*F. delavayi*	C_27_H_45_NO_2_	19	Zhou et al. (2010)
	Delavinone	*F. delavayi*	C_27_H_43_NO_2_	20	
	Imperiazine	Multiple botanical origins	C_27_H_43_NO_3_	21	Geng et al. (2018), Shakirova and Shakirov (2001)
	Zhebeinone	*F. thunbergii.* Rarely reported in Fritillariae Cirrhosae Bulbus	C_27_H_43_NO_3_	22	Geng et al. (2018), Zhang et al. (1992)
	Peimisine-3-O-β-D-glucopyranoside	*F. unibracteata*	C_33_H_51_NO_8_	23	Zhang et al. (2011)
	Sipeimine-3-O-β-D-glucopyranoside	*F. cirrhosa D. Don*	C_33_H_53_NO_8_	24	
	Yibeinoside A	*F. pallidiflora*	C_34_H_55_NO_6_	​	​
	Isoverticine	multiple botanical origins	C_27_H_45_NO_3_	​	​
	Songbeisine	To be confirmed	C_27_H_41_NO_3_	25	​
	Songbeinone	To be confirmed	C_27_H_43_NO_2_	26	​
	Songbeinine	To be confirmed	C_27_H_45_NO_2_	27	​
	Hupehenine	*F. delavayi*	C_27_H_45_NO_2_	28	Wang et al. (2021)

Total alkaloids refer to acid-base extracted, alkaloid-enriched fractions (purity 30%–60%) unless stated otherwise (Wang D. et al., 2016; Wang et al., 2011).

## 3 Pharmacological effects of alkaloids in Fritillariae Cirrhosae Bulbus

Although the alkaloid content in Fritillariae Cirrhosae Bulbus is relatively low (approximately 0.02%–0.3%) (Gu et al., 2026-04), it serves as a crucial indicator for quality assessment. Several alkaloids have shown cytotoxic activity against cancer cell lines *in vitro*, indicating potential anti-tumor effects that warrant further *in vivo* validation (Li et al., 2020; Kavandi et al., 2015). Wang D. et al. (2016) investigated the *in vivo* anti-inflammatory activity of total alkaloids from Fritillariae Cirrhosae Bulbus using various models. The results showed that the total alkaloids exhibited inhibitory effects on inflammation across all models, demonstrating potent anti-inflammatory activity. This anti-inflammatory effect may be associated with the inhibition of inflammatory mediators such as histamine and prostaglandins. Zhang et al. (2021) used LPS-stimulated RAW264.7 macrophages as an inflammation model to study the effect of sipeimine-3-glucoside on oxidative stress. The results indicated that sipeimine-3-glucoside could inhibit LPS-induced oxidative stress, suggesting that Fritillariae Cirrhosae Bulbus holds promise as a potential therapeutic agent for oxidative stress-related diseases. Liu et al. (2020) investigated the potential protective effects and mechanisms of six alkaloids from Fritillariae Cirrhosae Bulbus against cigarette smoke-induced oxidative stress in RAW 264.7 macrophages. The results showed that these six alkaloids could reduce reactive oxygen species (ROS) production and increase glutathione (GSH) levels, indicating that the alkaloids from Fritillariae Cirrhosae Bulbus may exert protective effects against cellular oxidative stress by activating the Nrf2-mediated antioxidant pathway.

### 3.1 Antitussive and antiasthmatic effects

The alkaloids from Fritillariae Cirrhosae Bulbus possess significant therapeutic effects in relieving cough, resolving phlegm, and alleviating dyspnea. The application of *Fritillaria* in treating cough and dyspnea has a millennia-long history in traditional Chinese medicine. Historical records, such as *Rihuazi Herba*l (Xin et al., 2022) and *General Collection* for Holy Relief (Xie et al., 2022), document its use for cough relief… Furthermore, the “Fritillariae Cirrhosae Bulbus Decoction” recorded in the General Collection for Holy Relief was specifically prescribed for treating coughs caused by wind-cold. Sun (2011) evaluated the antiasthmatic effect of a compound formula containing Fritillariae Cirrhosae Bulbus using a histamine-acetylcholine mixed aerosol-induced asthma model in guinea pigs and an isolated guinea pig tracheal spiral strip preparation. High (200 mg/kg) and medium (100 mg/kg) doses of the compound formula (administered intragastrically) significantly prolonged the asthma latent period. The results showed that both high and medium doses of the compound formula significantly prolonged the latent period of asthma induction and significantly reduced the contractile tension of isolated tracheal strips induced by acetylcholine, demonstrating a notable antiasthmatic effect. Yan et al. (2009) investigated the effects of ethanol extracts from three different species of Fritillariae Cirrhosae Bulbus on respiratory dynamics in a guinea pig model of allergic asthma. They found that all dose groups of the three extracts significantly inhibited the increase in airway resistance following allergen challenge, thereby exerting an antiasthmatic effect. Notably, no statistically significant difference was observed between the antiasthmatic effects of cultivated and wild varieties. Wang et al. (2011) studied the antitussive, expectorant, and anti-inflammatory effects of four alkaloids (peimine, peiminine, sipeimine, and chuanbeinone) isolated from Fritillariae Cirrhosae Bulbus using an ammonia-induced cough model in mice, a phenol red secretion model for evaluating expectorant activity, and a xylene-induced ear edema model in mice. The results indicated that all four alkaloids significantly suppressed the frequency of cough and prolonged the cough latent period in the ammonia-induced model. Among them, three alkaloids increased phenol red secretion, and two significantly inhibited the development of ear edema. Furthermore, the authors speculated that the presence of 17-βH, 22-αH, and 20-OH moieties might play important roles in mediating the antitussive, anti-inflammatory, and expectorant activities of these alkaloids. Yan et al. (2019) explored the effects of Fritillariae Cirrhosae Bulbus on Notch2 expression and the inflammatory response in lung tissue using an ovalbumen-sensitized mouse model of asthma. The results demonstrated that Fritillariae Cirrhosae Bulbus could reduce airway resistance, decrease the levels of certain inflammatory cytokines in serum, and downregulate Notch2 protein expression in the lung tissue of asthmatic mice. This suggests that the antiasthmatic effect of Fritillariae Cirrhosae Bulbus might be associated with the downregulation of Notch2 protein expression.

Currently, the therapeutic effects of Fritillariae Cirrhosae Bulbus on respiratory diseases are widely recognized within the international academic community. Research on the antitussive and antiasthmatic pharmacological activities of its alkaloids is relatively well-established in China, with some mechanisms now being explained at the level of protein regulatory pathways. However, internationally, studies specifically focusing on its antitussive and antiasthmatic effects are not a predominant research hotspot for Fritillariae Cirrhosae Bulbus, and relevant reports are relatively scarce. Further isolation and identification of its active metabolites, along with in-depth investigation of the specific mechanisms underlying their pharmacological activities, are required.

### 3.2 Antibacterial effects

Modern pharmacological studies indicate that various pharmacological activities of Fritillariae Cirrhosae Bulbus are closely related to its diverse bioactive metabolites, among which alkaloids are the primary substances exerting key pharmacological effects. Monomeric alkaloids (such as peimine, peiminine, ebeiedine, etc.,) showed specific antibacterial activities (Chen et al., 2017; Huang et al., 2017; Xiao et al., 1992) and are regarded as potential alternatives to antibiotics, showing broad application prospects in the field of animal feed additives.

As early as 1984, Xiong Wei et al. confirmed through bacteriostatic tests that the ethanol extract of Fritillariae Cirrhosae Bulbus significantly inhibited *Staphylococcus aureus* and *Escherichia coli* (Xiong et al., 1986). Xiao Canpeng et al. further demonstrated using filter paper disk susceptibility tests that monomeric alkaloids such as peimine, peiminine, and ebeiedine showed specific antibacterial activities against *Moraxella catarrhalis*, *Staphylococcus aureus*, *Escherichia coli*, and *Klebsiella pneumoniae*, although the bacteriostatic effects were relatively weak, their MIC values (6.25–200 μg/mL for bacteria (Bhat et al., 2022)) are higher than conventional antibiotics (e.g., ampicillin MIC = 0.5 μg/mL for *Escherichia coli*), but their low toxicity makes them suitable for long-term feed addition (Xiao et al., 1992).

Bhat et al. (2022) determined the minimum inhibitory concentrations (MICs) of Fritillariae Cirrhosae Bulbus extracts against six bacterial strains and 3 fungal strains using the broth microdilution method. The results showed MICs ranging from 6.25 to 200 μg/mL for bacteria and from 50 to 400 μg/mL for fungi. Huibo et al. (2000) found that *F. delavayi* exhibited strong inhibitory activity against *Streptococcus pneumoniae* (MIC: 125 μg/mL), while its inhibitory effects against *Haemophilus influenzae* and *Staphylococcus aureus* were comparatively weaker (MIC: 25 mg/mL).

Chen Jing et al. isolated five alkaloid-producing endophytic actinomycetes from *F. unibracteata*. Further assays revealed that the fermentation products contained isosteroidal alkaloids structurally similar to those from Fritillaria bulbs. Thin-layer chromatography combined with bioautography indicated a specific correlation between the types of alkaloids produced and their antibacterial activities (Chen et al., 2017). This finding aligns with previous research conclusions on the correlation between the metabolites of plant endophytes and their functions (Ju et al., 2015). This study elucidates the source of the antibacterial effects of Fritillariae Cirrhosae Bulbus alkaloids from a microecological perspective and also provides a potential pathway for the targeted production of its active metabolites using synthetic biology techniques. Currently, it is hypothesized that the antibacterial mechanism of Fritillariae Cirrhosae Bulbus alkaloids may involve disrupting bacterial cell membrane integrity or inhibiting key metabolic enzyme activities (Chen et al., 2017; Xiao et al., 1992); however, the specific targets and molecular pathways remain to be further elucidated.

### 3.3 Anti-inflammatory effects

The alkaloid metabolites present in Fritillariae Cirrhosae Bulbus possess significant anti-inflammatory activity. Research has confirmed that key active metabolites responsible for this anti-inflammatory effect within the alkaloid extracts include sipeimine, peimisine, and peimine (Wang et al., 2025). Huang Lijing et al. (Lijing et al., 2009) found that intragastric administration of an aqueous extract from *Fritillaria ussuriensis* (1–4 g kg^-1^) for five consecutive days dose-dependently reduced xylene-induced ear edema in mice and egg white-induced paw edema in rats, while also decreasing capillary permeability in mice. However, the anti-inflammatory efficacy of Fritillariae Cirrhosae Bulbus is notably influenced by its botanical origin and formulation. Huang Yabin et al. (Huang et al., 2018) compared the inhibitory effects of alkaloids from Songbei, Qingbei, Lubei, and *F. taipaiensis* (wild and cultivated. Alkaloids were not purified from these formulations; instead, the extracts were analyzed *via* HPLC-MS to confirm the presence of sipeimine, peimisine, and peimine) on xylene-induced ear edema in mice. The results showed that the inhibition rates of alkaloids from Qingbei and Lubei were significantly higher than those of the inhibition rates from *F. taipaiensis* (both wild and cultivated). Conversely, a study by Ma et al. (2014) indicated that the powder and ethanol extract of *F. taipaiensis* also effectively inhibited mouse ear edema and reduced swelling rates, demonstrating a clear anti-inflammatory effect.

At the molecular mechanism level, the anti-inflammatory action of Fritillariae Cirrhosae Bulbus involves multiple signaling pathways, including p53, IL-17, and TNF (Guo, 2023). The alkaloid metabolites primarily exert their anti-inflammatory effects by inhibiting the phosphorylation of MAPK signaling pathways, downregulating the expression of inflammatory mediators, and attenuating the transcriptional activity of the nuclear transcription factor NF-κB (Ling et al., 2020). Furthermore, studies have further revealed that metabolites such as sipeimine, peimisine, and peimine, found in the total alkaloids, can target and inhibit the JAK2-STAT3 signaling pathway. The three alkaloids are distinct from other components in the extracts and are the primary mediators of anti-inflammatory effects (Wang et al., 2025). This inhibition blocks the polarization process of M2-type macrophages, thereby significantly alleviating airway inflammation in an ovalbumen (OVA)-induced mouse model of asthma (Wang et al., 2025). This suggests that regulating macrophage polarization has become an important direction for elucidating the anti-inflammatory mechanism of Fritillariae Cirrhosae Bulbus.

### 3.4 Antioxidant effects

Oxidative damage is a biochemical process that disrupts the redox balance within cells due to an excess of intracellular oxidants (Hu et al., 2024). It is closely associated with various diseases and health issues. Consequently, the focus of antioxidant development research has increasingly shifted towards functional food ingredients (Nwozo et al., 2023). Numerous current studies indicate that *Fritillaria* species are safe, suitable for daily intake, and represent functional food ingredients rich in substances with high antioxidant activity (Zhang et al., 2024). In the cited study, a total alkaloid extract from *F. cirrhosa* (not a single alkaloid) reduced oxidative markers under hypoxic conditions *in vitro*. Zhu (2010), using a chronic hypoxia rat model, observed that Fritillariae Cirrhosae Bulbus could alleviate hypoxic stress by protecting the diaphragm against fatigue. This demonstrates that *Fritillaria* possesses anti-hypoxia effects, along with protective actions involving the reduction of lipid peroxidation and the scavenging of oxygen-free radicals. Research by Liu et al. (2020) found that under conditions of cigarette smoke-induced oxidative stress, isosteroidal alkaloids from Fritillariae Cirrhosae Bulbus exert antioxidant effects by reducing reactive oxygen species (ROS) generation, increasing glutathione (GSH) levels, and promoting the expression of heme oxygenase-1 (HO-1). The underlying mechanism may be associated with promoting Nrf2 nuclear translocation and upregulating Nrf2 expression. Furthermore, Peimine, peiminine, imperialine-3-O-β-D-glucopyranoside, delavine, and peimisine were tested individually, and their antioxidant activities were stronger than that of imperialine alone.

The aforementioned literature indicates that Fritillariae Cirrhosae Bulbus can alleviate oxidative stress by regulating the expression of key antioxidant proteins. Moreover, its various alkaloid metabolites exhibit significant differences in antioxidant activity. This provides an important basis for further elucidating the material basis and molecular mechanisms underlying its antioxidant effects.

### 3.5 Antitumor effects

Fritillariae Cirrhosae Bulbus, a traditional Chinese medicinal plant, has its dried bulbs widely used in the treatment of cough and asthma. In recent years, multiple studies have further indicated that Fritillariae Cirrhosae Bulbus possesses significant antitumor potential. Its total alkaloids, triterpenoids, and polysaccharides exhibit inhibitory activity against various tumor cells, including lung cancer, colon cancer, liver cancer, endometrial cancer, and ovarian cancer cells (Chen T. et al., 2020).

Research has shown that the total alkaloids exhibit potent antitumor activity (*in vivo*, 50 mg/kg intragastrically in mice) with low toxicity, while *in vitro* studies used concentrations of 10–50 μM. They can significantly inhibit tumor angiogenesis, induce cell apoptosis, and promote tumor cell apoptosis by activating caspase-3 (WANG et al., 2014). Studies by Zheng et al. (2016) have also confirmed that peiminine can significantly inhibit the growth of transplanted HCT-116 tumors in mice by inducing apoptosis and autophagy. It can also promote autophagic flux and apoptotic processes by regulating levels of metabolites such as glucose, glutamine, and oleic acid, leading to the death of HCT-116 cells (Zheng et al., 2017). Previous research has also pointed out that the total alkaloids of Fritillariae Cirrhosae Bulbus and various monomeric metabolites thereof, such as chuanbeinone, imperialine, imperialine N-oxide, isoverticine, and its N-oxide, and peimisine, can effectively inhibit the proliferation of eukaryotic tumor cells. Moreover, at equivalent concentrations, the inhibitory effects of these metabolites were superior to those of peimine and peiminine (Sichuan University and Chengdu West China Natural Medicine Co, 2012). Research by Zhang et al. (2016) indicated that peiminine primarily inhibits cell proliferation and induces apoptosis in colon cancer HCT-116 cells by interfering with the metabolic pathway of fluorouracil. Additionally, peiminine can enhance the sensitivity of various human tumor cell lines, including esophageal cancer Eca-109, breast cancer MCF-7, non-small cell lung cancer A549, liver cancer HepG2, and cervical cancer HeLa, to the chemotherapeutic agent doxorubicin, suggesting its potential as a novel chemosensitizer (Tang et al., 2017).

### 3.6 Antihypertensive effects

Research on the antihypertensive effects of alkaloid metabolites from Fritillariae Cirrhosae Bulbus is currently limited. However, existing evidence suggests that its alkaloids possess the potential to relax blood vessels and lower blood pressure, showcasing specific application prospects (Na, 2025). Studies indicate (Kang et al., 2004; Kang et al., 2002) that extracts of Fritillariae Cirrhosae Bulbus can achieve antihypertensive effects by inhibiting angiotensin-converting enzyme (ACE) activity, promoting the release of the NO/cGMP signaling pathway, increasing vascular NO supply, regulating plasma NO metabolite concentrations, and concurrently improving renal function. Research by Zhaoli et al. (2023) further confirmed that alkaloid extracts from Fritillariae Cirrhosae Bulbus can alleviate hypertensive symptoms, improve vascular and myocardial function, reduce blood lipid levels, and exert renal protective effects in spontaneously hypertensive rats (SHRs). The antihypertensive mechanism primarily involves regulating nicotinate and nicotinamide metabolism, influencing the renin-angiotensin system (RAS), and maintaining the nitric oxide-endothelin (NO-ET) balance, thereby significantly reducing pathological damage caused by hypertension. Although this study has not fully elucidated the specific pathways involved, it still provides a new research direction for the prevention and treatment of hypertension and its related complications.

### 3.7 Other pharmacological effects

#### 3.7.1 Analgesic and sedative effects

Studies have shown that alkaloids derived from Fritillariae Cirrhosae Bulbus possess definite analgesic activity. Among these, the isosteroidal alkaloid verticinone significantly inhibits acetic acid-induced pain responses in mice. Experimental data indicate that verticinone at a concentration of 3 mg/kg exhibited superior analgesic effects compared to 200 mg/kg of aspirin. In neuropathic pain models, the analgesic effect of verticinone lasts longer and is more stable than that of morphine (Xu et al., 2011). Another alkaloid metabolite from Fritillariae Cirrhosae Bulbus, peimine, exerts analgesic effects by blocking voltage-gated sodium channels such as Nav1.7 (Xu et al., 2016; Yin et al., 2019). Nav1.7, a key threshold channel for pain signal transmission, is an important target for pain therapy. It is primarily distributed in peripheral sensory neurons and sympathetic ganglion neurons, where its activation can amplify low-intensity stimuli, leading to excitation of the dorsal root ganglia (DRG) and transmission of pain signals (Wood et al., 2019). Qian and Hengjun (1985) found that peimine and peiminine also possess sedative effects on the central nervous system. At a concentration of 2.0 mg/kg, both metabolites reduced spontaneous activity in mice as well as increased activity induced by caffeine, prolonged sleep time, and increased the rate of sleep onset, thereby exhibiting central inhibitory effects.

#### 3.7.2 Antidiabetic effects

Research indicates that peimisine exhibits inhibitory activity against dipeptidyl peptidase-IV (DPP-4), demonstrating promising therapeutic effects in diabetes treatment (Gu et al., 2019). DPP-4 inhibition primarily improves symptoms of type 2 diabetes mellitus by increasing endogenous glucagon-like peptide-1 (GLP-1) levels, which in turn promotes insulin release and suppresses glucagon secretion (Gallwitz, 2019). Furthermore, another alkaloid metabolite, peiminine, has been shown to exert antidiabetic effects *in vitro* experiments by modulating the function of pancreatic β-TC6 cells and C2C12 skeletal muscle cells (Boojar et al., 2020).

#### 3.7.3 Antimalarial activity

Studies have confirmed that the chloroform extract of Fritillariae Cirrhosae Bulbus, as well as its contained alkaloid marker metabolites (such as peimine, peiminine, puqiedinone, and puqiedine), exhibit significant inhibitory activity against drug-resistant Plasmodium falciparum (Bora et al., 2023). Pf-CRT is a transmembrane protein that mediates drug efflux in drug-resistant Plasmodium falciparum. Alkaloids bind to Pf-CRT, inhibiting its efflux function and restoring susceptibility to antimalarials. The effects were observed at concentrations of 10–25 μM. This discovery not only provides a scientific basis for the application of Fritillariae Cirrhosae Bulbus in malaria prevention and treatment but also establishes a theoretical foundation for further research and development of its metabolites as novel antimalarial lead compounds or metabolites in combination therapy strategies.

## 4 Conclusion

This review systematically summarizes the major types of alkaloids found in Fritillariae Cirrhosae Bulbus and their diverse pharmacological activities. It clarifies that the alkaloids from Fritillariae Cirrhosae Bulbus encompass hundreds of compounds, including steroidal, isosteroidal, and other types. Among these are isosteroidal alkaloids such as peimine, peiminine, and imperialine, which are identified as key metabolites responsible for its pharmacological effects. Regarding pharmacological activities, the alkaloids not only embody the core traditional Chinese medicinal effects of relieving cough, alleviating dyspnea, dissipating nodules, and reducing abscesses, but their antibacterial, anti-inflammatory, antioxidant, antitumor, and antihypertensive properties have also been well-validated. In human clinical applications, alkaloids from Fritillariae Cirrhosae Bulbus mainly exert antitussive, antitumor, antihypertensive, and antimalarial activities. Although no direct veterinary clinical trials have been reported to date, the documented antitussive, anti-inflammatory, antibacterial, and antioxidant activities suggest that alkaloids from Fritillariae Cirrhosae Bulbus hold promise for future animal clinical applications. Further studies in target animal species are warranted. Processing by-products can be used as feed additives. Furthermore, their potential value in areas such as analgesic-sedative, antidiabetic, and antimalarial applications is gradually being confirmed.

However, numerous unresolved issues that urgently require breakthroughs still exist in current research, which are mainly manifested in the following aspects. First, the specific active ingredients responsible for the pharmacological effects have not been accurately identified. Some studies only regard “total alkaloids” as the active fraction for efficacy evaluation of crude extracts, without further isolating and identifying the key monomeric metabolites that play a crucial role. The extraction methods of alkaloids reported in some studies are summarized in Table 2. This deficiency has resulted in difficulties in the horizontal comparison of results among different studies. Second, some pharmacological mechanisms and action targets remain unclear. Most studies merely propose that the pharmacological effects of Fritillariae Cirrhosae Bulbus alkaloids are associated with a certain signaling pathway, but fail to conduct in-depth investigations or elaborate on the specific action targets. Third, the influences of different botanical origins and dosage forms on pharmacological efficacy remain unclear. As recorded in the Chinese Pharmacopoeia, Fritillariae Cirrhosae Bulbus has multiple botanical origins, including *F. unibracteata, F. taipaiensis,* and *Fritillaria wabuensis*. However, the types and contents of alkaloids vary significantly among these different origins, and comparative studies on the equivalence of their anti-inflammatory or anti-tumor effects are still lacking. Additionally, there is no systematic report on whether different dosage forms exert varying effects on the pharmacological efficacy. In summary, future research should prioritize the identification of the effects of specific alkaloid species on a particular pharmacological activity. It is necessary to conduct in-depth studies on the specific action targets of relevant signaling pathways to fill the gaps in mechanistic research. Furthermore, standardized methods for the extraction and characterization of Fritillariae Cirrhosae Bulbus alkaloids (in accordance with the ConPhyMP guidelines) should be established to improve the reproducibility of research results, thereby distinguishing the differences in pharmacological efficacy of alkaloids from different botanical origins of Fritillariae Cirrhosae Bulbus.

**TABLE 2:** Summary of extract characteristics in referenced studies.

References	Extract type	Extraction solvent	Extraction method	Preparation process
Liu et al. (2020)	Isosteroid alkaloids	Ethanol/methanol (basified with ammonia water); acetonitrile, methanol, water, and base-saturated diethyl ether are used for purification	Basified infiltration with ammonia water, reflux/ultrasonic extraction with ethanol, followed by resin purification and extraction separation	Dried and pulverized Fritillariae Cirrhosae Bulbus → infiltration with ammonia water → ethanol extraction → filtration and concentration → resin purification → volume adjustment
Wang et al. (2016a)	Total alkaloid fraction	The specific extraction solvent was not reported, and H-103 macroporous resin was mainly employed for purification	H-103 resin column chromatography	Extraction from Fritillariae Cirrhosae Bulbus bulbs → purification on an H-103 macroporous resin column → preparation of the total alkaloid fraction
Bhat et al. (2022)	Crude extracts of Fritillaria cirrhosa bulbs	Methanol, petroleum ether, ethyl acetate	Cold/room temperature extraction (crude extract preparation) + LC-MS analysis	Fritillaria cirrhosa bulbs → crude extraction with methanol/petroleum ether/ethyl acetate → LC-MS phytoconstituent analysis
Zhang et al. (2021)	Edpetiline	Not specified	Isolation and purification of alkaloids	Fritillariae Cirrhosae Bulbus → pulverization → extraction → purification → edpetiline monomer
Sun (2011)	Fritillariae Cirrhosae Bulbus Antitussive Granules	Not specified	Not specified	Medicinal herb compatibility → extraction and concentration → granulation and forming
Yan et al. (2009)	Alcohol extracts of wild *Fritillaria cirrhosa*, *Fritillaria wabuensis,* and *Fritillaria densiflora*	Ethanol	Ethanol reflux extraction	Fritillaria bulb pulverization → ethanol reflux extraction → filtration and concentration → alcohol extract obtained
Wang et al. (2011)	imperialine, chuanbeinone, verticinone, verticine	Ethanol	Phytochemical isolation and purification	ulbs of Fritillariae Cirrhosae → ethanol extraction → Isolation and purification → Individual alkaloid monomers
Yan et al. (2019)	Total alkaloids	Not specified	Not specified	Fritillariae Cirrhosae Bulbus medicinal material → Crushing → Extraction → Concentration → Dosing formulation (for intragastric administration or intraperitoneal injection)
Chen et al. (2017)	Alkaloid extract from the fermentation broth of endophytic actinomycetes isolated from *Fritillaria unibracteata* var. *Sichuanica*	Methanol, ethyl acetate	Fermentation broth extraction → Methanol immersion → Ethyl acetate extraction → Concentration under reduced pressure	Bulbs of *Fritillaria unibracteata* → Surface disinfection → Tissue isolation → Culture in modified Gause’s No. 1 medium → Screening of alkaloid-producing strains → Fermentation culture → Methanol immersion for 24 h → Ethyl acetate extraction 2–3 times → Concentration under reduced pressure at 60 °C → Dilution to volume with ethanol → Alkaloid extract
Xiao et al. (1992)	*Fritillaria* alkaloid monomers	Ethanol	Ethanol reflux extraction, followed by column chromatography separation and purification	*Fritillaria* bulbs are crushed → ethanol reflux extraction → concentration → column chromatography separation → obtainment of individual alkaloid monomers
Huibo et al. (2000)	Total alkaloid extracts from *Fritillaria pallidiflora* and *Fritillaria delavayi*	Ethanol	Ethanol reflux extraction	*Fritillaria* bulbs are crushed → ethanol reflux extraction → concentration → extract
Xiong et al. (1986)	Total alkaloid extract from *Fritillaria hupehensis*	Ethanol	Ethanol reflux extraction	*Fritillaria* bulbs are crushed → ethanol reflux extraction → concentration → extract
Wang et al. (2025)	Steroidal alkaloids/total alkaloids	Ethanol	Ethanol extraction, followed by resin enrichment and purification	*Fritillaria* bulbs are crushed → ethanol extraction → filtration and concentration → resin purification → alkaloid extract
Lijing et al. (2009)	Aqueous extract of *Fritillaria ussuriensis*	Water	Decoction extraction (water decoction)	*Fritillaria ussuriensis* bulbs are crushed → water decoction → filtration and concentration → obtainment of aqueous extract
Huang et al. (2018)	Total alkaloids from different varieties of *Fritillariae Cirrhosae* Bulbus	Ethano	Ethanol reflux extraction	*Fritillariae Cirrhosae* Bulbus is crushed → ethanol reflux extraction → concentration and purification → total alkaloids
Guo (2023)	Isosteroidal alkaloids of *Fritillariae Cirrhosae* Bulbus from Qinghai	70% ethanol (for experimental validation); virtual screening from databases for network pharmacology	Ethanol reflux extraction (experimental); TCMSP/ETCM database screening combined with ADME/T activity screening (computational)	*Fritillariae Cirrhosae* Bulbus is crushed → reflux extraction with 70% ethanol → filtration, concentration, and drying to obtain crude extract; the computational component involves target prediction, network construction, and molecular docking to elucidate the mechanism of action
Ling et al. (2020)	Polysaccharides from *Fritillaria ussuriensis*	Water	Ultrasound-assisted extraction	*Fritillaria ussuriensis* bulbs are crushed → ultrasound-assisted water extraction → concentration and purification → polysaccharide extract
Zhu (2010)	Extract of *Fritillariae Cirrhosae* Bulbus	Water	Decoction extraction	*Fritillariae Cirrhosae* Bulbus is crushed → water decoction → filtration and concentration → obtainment of the extract
Wang et al. (2014), Gu et al. (2019), Boojar et al. (2020), Bora et al. (2023)	Total steroidal alkaloids from the bulbs of cultivated *Fritillariae Cirrhosae* Bulbus	Ethanol	Extraction optimized by orthogonal test, followed by enrichment and purification with H-103 macroporous resin	Bulbs of *Fritillariae Cirrhosae* Bulbus are crushed → ethanol extraction → filtration and concentration → adsorption and elution with H-103 resin → purification and enrichment of alkaloids
Zheng et al. (2016), Zheng et al. (2017), Zhang et al. (2016), Tang et al. (2017), XU et al. (2011)	Peiminine	Ethanol	Ethanol reflux extraction, followed by column chromatography separation and purification	Bulbs of *Fritillaria thunbergii* are crushed → ethanol reflux extraction → concentration → column chromatography separation → purification to obtain peiminine monomer
Sichuan University and Chengdu West China Natural Medicine Co (2012)	Steroidal alkaloids	Methanol/ethanol (organic solvents), chloroform-methanol mixture, chloroform-ethanol mixture, acidic water (containing 0.5% HCl), etc	Reflux extraction, purification by acid-solubilization and alkali-precipitation, enrichment by chloroform extraction	*Fritillariae Cirrhosae* Bulbus is crushed → organic solvent reflux extraction→ concentration under reduced pressure → kneading and dissolution with 3% HCl → defatting with petroleum ether → adjustment of pH to 10 with ammonia water → extraction of alkaloids with chloroform → vacuum drying at 45 °C → obtainment of total alkaloids from *Fritillariae Cirrhosae* Bulbus
(Kang et al., 2004; Kang et al., 2002)	Aqueous extract of *Fritillaria* bulbs (BFWE)	Water	Decoction extraction	*Fritillaria* bulbs are crushed → water decoction → filtration and concentration → obtainment of aqueous extract
Zhaoli et al. (2023)	Total alkaloids of *Veratrum*, Fraction A, veratramine	Ethanol	Ethanol reflux extraction, separation, and purification	*Veratrum* is crushed → ethanol reflux extraction → concentration → purification to obtain the products
Qian and Hengjun (1985)	Verticine and verticinone	Ethanol	Ethanol extraction, followed by column chromatography separation and purification	*Fritillaria thunbergii* is crushed → ethanol extraction → concentration → chromatographic purification → individual monomers

In summary, by integrating existing research findings, this review establishes a clear framework for understanding the types of alkaloids from Fritillariae Cirrhosae Bulbus, their potential pharmacological effects, and the current issues and challenges in related research. It aims to provide research directions for the further development and clinical application of these alkaloids in animal production, thereby contributing to the steady advancement of modernization research on Fritillariae Cirrhosae Bulbus.

## References

[B1] Bhat B. A. Mir W. R. Sheikh B. A. Alkanani M. Mir M. A. (2022). Metabolite fingerprinting of phytoconstituents from *Fritillaria cirrhosa D. Don* and molecular docking analysis of bioactive peonidin with microbial drug target proteins. Sci. Rep. 12 (1), 7296. 10.1038/s41598-022-10796-7 35508512 PMC9068770

[B2] Boojar F. M. A. Aghaei R. Mashhadi Akbar Boojar M. (2020). Data on possible *in vitro* anti-diabetic effects of verticinone onβ-TC6 pancreatic and C2C12 skeletal muscle cells. Data Brief. 28, 104828. 10.1016/j.dib.2019.104828 31867414 PMC6906668

[B3] Bora P. S. Agrawal P. Kaushik N. K. Puri S. Sahal D. Sharma U. (2023). Antiplasmodial activity of the bulbs of *Fritillaria cirrhosa D.Don* (Syn: *Fritillaria roylei Hook*.): UPLC-IM-Q-TOF-MS/MS-based biochemometric approach for the identification of marker compounds. J. Ethnopharmacology 310, 116389. 10.1016/j.jep.2023.116389 36924862

[B4] Cao X. (2008). Study on chemical metabolites of *Fritillaria cirrhosa* and quality evaluation of medicinal plants of the *Fritillaria* genus. Peking Union Medical College.

[B5] Chang H. C. Xie H. M. Lee M. R. Lin C. Y. Yip M. K. Agrawal D. C. (2020). *In vitro* propagation of bulblets and LC-MS/MS analysis of isosteroidal alkaloids in tissue culture derived materials of Chinese medicinal herb *Fritillaria* cirrhosa D. Don. Bot. Stud. 61 (1), 9. 10.1186/s40529-020-00286-2 32211983 PMC7093630

[B6] Chen J. Xian-Ci-Zu T. Hong Z. Zeng-Yan L. Fang W. Song-Qing L. (2017). Screening, identification, and antimicrobial activity of alkaloids produced by endophytic actinomycetes from *Fritillaria unibracteata* in western Sichuan plateau. China J. Chin. Materia Medica 42 (23), 4582–4587. 10.19540/j.cnki.cjcmm.20171030.013 29376255

[B7] Chen Y. Guo S. Guan Y. Li M. An Y. Liu H. (2019). The research progress of medicinal plants *fritillaria* . Mol. Plant Breed. 17 (18), 6198–6206.

[B8] Chen C. C. Lee M. R. Wu C. R. Ke H. J. Xie H. M. Tsay H. S. (2020a). LED lights affecting morphogenesis and isosteroidal alkaloid contents in *Fritillaria cirrhosa D. Don*-an important Chinese medicinal herb. Plants (Basel) 9 (10). 10.3390/plants9101351 33066243 PMC7602057

[B9] Chen T. Zhong F. Yao C. Chen J. Xiang Y. Dong J. (2020b). A systematic review on traditional uses, sources, phytochemistry, pharmacology, pharmacokinetics, and toxicity of fritillariae cirrhosae bulbus. Evid. Based Complement. Altern. Med. 2020, 1536534. 10.1155/2020/1536534 33273948 PMC7676930

[B10] Cui Z. Ma Y. Zhang X. Shao J. Jin L. Ma Y. (2021). Research progress on chemical components and pharmacological effects and predictive analysis on quality markers of Fritillariae Cirrhosae Bulbus. Chin. Traditional Herb. Drugs 52 (9), 2768–2784.

[B11] Cunningham A. B. Brinckmann J. A. Pei S. J. Luo P. Schippmann U. Long X. (2018). High altitude species, high profits: can the trade in wild harvested *Fritillaria cirrhosa* (Liliaceae) be sustained? J. Ethnopharmacol. 223, 142–151. 10.1016/j.jep.2018.05.004 29751123

[B12] Gallwitz B. (2019). Clinical use of DPP-4 inhibitors. Front. Endocrinol:Lausanne 10, 389. 10.3389/fendo.2019.00389 31275246 PMC6593043

[B13] Geng Z. Liu Y. F. Gou Y. Zhou Q. He C. Guo L. (2018). Metabolomics study of cultivated bulbus *fritillariae cirrhosae* at different growth stages using UHPLC-QTOF-MS coupled with multivariate data analysis. Phytochem. Anal. 29 (3), 290–299. 10.1002/pca.2742 29336082

[B14] Gu L. H. Tian T. Xia L. Chou G. Wang Z. (2019). Rapid isolation of a dipeptidyl peptidase IV inhibitor from *Fritillaria cirrhosa* by thin-layer chromatography-bioautography and mass spectrometry-directed autopurification system. JPCJ Planar Chromatogr-Mod TLC 32 (6), 447–451. 10.1556/1006.2019.32.6.1

[B15] Gu S. M. Yin J. Shu Q. Li L. Li H. (2026). A review on germplasm resources and quality evaluation of the rare and endangered *fritillaria cirrhosa* bulbus [J/OL]. North. Hortic. 1-19.

[B16] Guo J. (2023). Study on the anti-inflammatory mechanism of active metabolites from Qinghai *Fritillaria cirrhosa* based on network pharmacology and molecular docking [D]. Qinghai Normal University. 10.27778/d.cnki.gqhzy.2023.000419

[B17] Guo Y. Xie H. Xie H. Fu S. Wang N. Wang S. (2019). Comparison of the appearance characters of the cultivated and wild *Fritillariae cirrhosae* Bulbus. West China J. Pharm. Sci. 34 (06), 613–616.

[B18] Han G. Qi L. He D. Xue W. Li W. Wang H. (2025). Multidimensional quality evaluation and traceability study of Fritillariae Cirrhosae Bulbus from different sources. Front. Plant Sci. 16, 1648434. 10.3389/fpls.2025.1648434 41019711 PMC12460310

[B19] Hou C. Lu X. Lei X. Tang Y. Zhao R. Li B. (2023). Effect of *Fritillaria cirrhosa* on OVA-sensitized asthmatic mice and its possible mechanism. Med. J. Chin. People's Liberation Army 1-10–20.

[B20] Hu B. Ouyang Y. Zhao T. Wang Z. Yan Q. Qian Q. (2024). Antioxidant hydrogels: antioxidant mechanisms, design strategies, and applications in the treatment of oxidative stress-related diseases. Adv. Healthcare Materials 13 (11), e2303817. 10.1002/adhm.202303817 38166174

[B21] Huang C. Chen F. Peng J. Liu X. Yu Y. Tang W. (2017). Effect of *Fritillaria cirrhosa* on the formation of biofilm of *Escherichia coli* . Chin. J. Nosocomiology 27 (22), 5049–5052.

[B22] Huang Y. Liu H. Fang C. Yu Y. Chen H. Zhang S. (2018). Comparative study on the pharmacodynamic differences of the anti-tussive and anti-inflammatory effects of the alkaloids from different varieties of fritillariae cirrhosae bulbus. Traditional Chin. Drug Res. and Clin. Pharmacol. 29 (01), 19–22. 10.19378/j.issn.1003-9783.2018.01.004

[B23] Huang M. Wu H. Yu W. Wang Y. Wang F. Zhang C. (2021). Rapid identification of chemical components in Qi-Yu-San-Long decoction by ultra high performance liquid chromatography-quadrupole time-of-flight mass spectrometry. Chin. J. Chromatogr. 39 (07), 730–743. 10.3724/SP.J.1123.2020.10016 PMC9404180 34227371

[B24] Huibo Xu Sun X. Wen F. Zhou J. Ding T. Sun L. (2000). A preliminary comparative study on physiological activity of *Fritillaria pallidiflora schrek*. And F. Delavayi Franch. China J. Chin. Materia Medica (07), 7–9. 12515218

[B25] Jiang S. Sun H. Qin J. Zhu W. T. (2016). Functional production regionalization for Fritillariae Cirrhosae Bulbus based on growth and quality suitability assessment. China J. Chin. Materia Medica 41 (17), 3194–3201. 10.4268/cjcmm20161713 28920370

[B26] Ju C. Zhang X. Jiangyuan Z. Wen M. Ding Z. Li M. (2015). The recent progress of study on secondary metabolites of Streptomyces. Chin. J. Antibiotics 40 (10), 791–800. 10.13461/j.cnki.cja.005629

[B27] Kang D. G. Oh H. Cho D. K. Kwon E. K. Han J. H. Lee H. S. (2002). Effects of bulb of *Fritillaria ussuriensis* maxim.on angiotensin converting enzyme and vascular release of NO/cGMP in rats. J. Ethnopharmacol. 81 (1), 49–55. 10.1016/s0378-8741(02)00037-5 12020927

[B28] Kang D. G. Sohn E. J. Yun M. L. Lee A. S. Han J. H. Kim T. Y. (2004). Effects of Bulbus *Fritillaria* water extract on blood pressure and renal functions in the L-NAME-induced hypertensive rats. J. Ethnopharmacol. 91 (1), 51–56. 10.1016/j.jep.2003.11.015 15036467

[B29] Kavandi L. Lee L. R. Bokhari A. A. Pirog J. E. Jiang Y. Ahmad K. A. (2015). The Chinese herbs Scutellaria baicalensis and *Fritillaria cirrhosa* target NFκB to inhibit proliferation of ovarian and endometrial cancer cells. Mol. Carcinog. 54 (5), 368–378. 10.1002/mc.22107 24249479

[B30] Li R. Zhang Y. Wang Y. Huang K. Yang Q. Zhang T. (2020). Aqueous extract of *Fritillariae cirrhosae* induces cellular apoptosis through activation of STATs-mediated immunomodulation. J. Ethnopharmacol. 261, 112338. 10.1016/j.jep.2019.112338 31669666

[B31] Lijing H. Gao W. Xia L. Zhang Y. (2009). Anti-inflammatory effects of aqueous extract of bulbus *fritillariae ussuriensis* . Tianjin J. Traditional Chin. Med. 26 (06), 495–496.

[B32] Ling T. Xie J. Shen Y. S. Qiao M. Yang H. Sun D. Y. (2020). Trichostatin A exerts anti-inflammation functions in LPS-induced acute lung injury model through inhibiting TNF-α and upregulating microRNA-146a expression. Eur. Review Medical Pharmacological Sciences 24 (7), 3935–3942. 10.26355/eurrev_202004_20861 32329869

[B33] Liu X. Qin Q. Soliman M. Cheng D. Liangwenyao Z. Linli C. (2019). Study on comprehensive quality of *fritillaria cirrhosa* . J. Chengdu Univ. Nat. Sci. Ed. 39 (03), 241–245.

[B34] Liu S. Yang T. Ming T. W. Gaun T. K. W. Zhou T. Wang S. (2020). Isosteroid alkaloids from Fritillaria cirrhosa bulbus as inhibitors of cigarette smoke-induced oxidative stress. Fitoterapia 140, 104434. 10.1016/j.fitote.2019.104434 31760067

[B35] Lu Q. Li R. Liao J. Hu Y. Gao Y. Wang M. (2022). Integrative analysis of the steroidal alkaloids distribution and biosynthesis of bulbs Fritillariae Cirrhosae through metabolome and transcriptome analyses. BMC Genomics 23 (1), 511. 10.1186/s12864-022-08724-0 35836113 PMC9284883

[B36] Ma P. Wang L. Wang Y. Peng R. (2014). Effects of *Taibai fritillaria* for relieving cough, expelling phlegm, and anti-inflammatory. Pharmacol. Clin. Chin. Materia Medica 30 (01), 87–89. 10.13412/j.cnki.zyyl.2014.01.029

[B37] Na Z. (2025). The “cough-relieving miracle” *Fritillaria cirrhosa* . Food Health 37 (9), 10–11.

[B38] Nwozo O. S. Effiong E. M. Aja P. M. Awuchi C. G. (2023). Antioxidant, phytochemical, and therapeutic properties of medicinal plants: a review. Int. J. Food Prop. 26 (1), 359. 10.1080/10942912.2022.2157425

[B39] Peng C. Jin P. Xiaofang X. Liang X. Min L. Shu W. (2022). Research ideas and practice on the application of key technologies for quality control and industrialization of genuine medicinal materials produced in Sichuan. Traditional Chin. Drug Res. and Clin. Pharmacol. 13 (03), 1–6.

[B40] Qian B. Hengjun X. (1985). Antitussive and sedative effects of peimine and peiminine. Acta Pharm. Sin. (04), 306–308. 10.16438/j.0513-4870.1985.04.013 4072703

[B41] Shakirova U. T. Shakirov R. (2001). Imperiazine, a new alkaloid from Petilium eduardi. Chem. Nat. Compd. 37 (5), 474–475. 10.1023/a:1014483729450

[B42] Shiwei Li (2022). Study on monosaccharide composition analysis of 52 kinds of polysaccharides from traditional Chinese medicine and quality evaluation of polysaccharides derived from Fritillarias. Changchun Univ. Chin. Med.

[B43] Sichuan University, Chengdu West China Natural Medicine Co (2012). New anticancer use of total alkaloids from *Fritillaria cirrhosa* and compounds contained therein–01-25. CN201110297762.9[P].

[B44] Sun T. (2011). Study on the mechanism of action of Chuanbeimu Zhike Granules in treating acute bronchitis [D]. Chengdu University of Traditional Chinese Medicine.

[B45] Tang Q. Wang Y. Nie Y. Gu Z. (2017). Study on the effect of peiminine on increasing the chemosensitivity of 5 kinds of cancer cells. China Pharm. 28 (34), 4796–4800.

[B46] Wang X. J. Li Y. M. (2013). Analysis of volatile oil of *Fritillaria cirrhosa D. Don* by GC-MS. Asian J. Chem. 25 (6), 3252–3254. 10.14233/ajchem.2013.13617

[B47] Wang D. D. Zhu J. Y. Wang S. Wang X. Ou Y. Wei D. (2011). Antitussive, expectorant, and anti-inflammatory alkaloids from bulbus *fritillariae cirrhosae* . Fitoterapia 82 (8), 1290–1294. 10.1016/j.fitote.2011.09.006 21958967

[B48] Wang D. Wang S. Feng Y. Zhang L. Li Z. Ma J. (2014). Antitumor effects of Bulbus *Fritillariae cirrhosae* on Lewis lung carcinoma cells *in vitro* and *in vivo* . Ind. Crops Prod. 54 (02), 92–101. 10.1016/j.indcrop.2013.12.054

[B49] Wang D. Yang J. Du Q. Li H. Wang S. (2016a). The total alkaloid fraction of bulbs of *Fritillaria cirrhosa* displays anti-inflammatory activity and attenuates acute lung injury. J. Ethnopharmacol. 193, 150–158. 10.1016/j.jep.2016.08.009 27497638

[B50] Wang D. D. Li Z. Zhang L. Atanasov A. G. Wang S. (2016b). Characterization of the isosteroidal alkaloid chuanbeinone from bulbus of *Fritillaria pallidiflora* as novel antitumor agent *in vitro* and *in vivo* . Planta Médica 82 (3), 195–204. 10.1055/s-0035-1558156 26584458

[B51] Wang D. Chen X. Atanasov A. G. Yi X. Wang S. (2017). Plant resource availability of medicinal *fritillaria* species in traditional producing regions in qinghai-tibet plateau. Front. Pharmacol. 8, 502. 10.3389/fphar.2017.00502 28824427 PMC5545572

[B52] Wang Y. Hou H. Ren Q. Hu H. Yang T. Li X. (2021). Natural drug sources for respiratory diseases from *Fritillaria*: chemical and biological analyses. Chin. Med. 16 (1), 40. 10.1186/s13020-021-00450-1 34059098 PMC8165352

[B53] Wang Y. Peng M. Yang X. Tu L. Liu J. Yang Y. (2025). Total alkaloids in *Fritillaria cirrhosa* D. Don alleviate OVA-induced allergic asthma by inhibiting M2 macrophage polarization. J. Ethnopharmacology 337 (Pt 3), 118935. 10.1016/j.jep.2024.118935 39396718

[B54] Wood J. Cox J. Zhao J. (2019). How Nav1.7 contributes to pain pathways. IBRO Rep. 6, S26. 10.1016/j.ibror.2019.07.070

[B55] Wu X. Chan S. W. Ma J. Li P. Shaw P. C. Lin G. (2018). Investigation of association of chemical profiles with the tracheobronchial relaxant activity of Chinese medicinal herb Beimu derived from various *Fritillaria* species. J. Ethnopharmacol. 210, 39–46. 10.1016/j.jep.2017.08.027 28842340

[B56] Xiao C. Haoru Z. Ping L. Guojun X. (1992). *In vitro* antibacterial activity of several main metabolites of traditional Chinese medicine Beimu. J. China Pharm. Univ. (03), 188–189.

[B57] Xiao T. Yang M. H. Wang Q. L. Lyu Q. Zheng Y. Q. Xu L. C. (2025). Domestication progress of endangered Chinese medicinal material Fritillariae Cirrhosae Bulbus. China J. Chin. Materia Medica 50 (16), 4483–4489. 10.19540/j.cnki.cjcmm.20250422.101 41084466

[B58] Xie J. Peng T. Lu H. Yu X. Qingmao F. Junning Z. (2022). Development status, strategies and methods of Fritillariae Cirrhosae Bulbus industrial chain based on generalized science of Chinese materia medica. Chin. Traditional Herb. Drugs 53 (07), 2150–2163.

[B59] Xin J. Chunnan L. Hui Z. (2022). Research progress on alkaloid metabolites and their pharmacological activities in *Fritillaria* medicinal materials. J. Chin. Med. Mater. (09), 2262–2268.

[B60] Xiong W. Guo X. He J. (1986). Preliminary study on pharmacological effects of *Fritillaria hupehensis* . Chin. Traditional Herb. Drugs 17 (03), 19–22. CNKI:SUN:ZCYO.0.1986-03-010.

[B61] Xu F. Xu S. Wang L. Chen C. Zhou X. Lu Y. (2011). Antinociceptive efficacy of verticinone in murine models of inflammatory pain and paclitaxel induced neuropathic pain. Biol. Pharm. Bull. 34 (09), 1377–1382. 10.1248/bpb.34.1377 21881221

[B62] Xu J. Zhao W. Pan L. Zhang A. Chen Q. Xu K. (2016). Peimine, a main active ingredient of *Fritillaria,* exhibits anti-inflammatory and pain suppression properties at the cellular level. Fitoterapia 111, 1–6. 10.1016/j.fitote.2016.03.018 27033404

[B63] Yan X. Meng X. Xiao H. Mo Z. (2009). Effect of three kinds of *Fritillaria cirrhosa* on respiratory dynamics in asthmatic Guinea pigs. China J. Chin. Materia Medica 34 (20), 2655–2659.

[B64] Yan W. Dapeng F. Sun L. Baohui Z. (2019). Effect of *fritillariae cirrhosae* bulbus on Notch2 and inflammatory response in asthmatic mice. Prog. Anatomical Sci. 25 (05), 583–585+589.

[B65] Yin Z. Zhang J. Guo Q. Chen L. Zhang W. Kang W. (2019). Pharmacological effects of verticine: current status. Evidence-based Complementary Alternative Medicine eCAM 2019, 2394605. 10.1155/2019/2394605 30956677 PMC6431433

[B66] Zhang J. Aina L. Huang H. Ma G. Xu R. (1992). Studies on the chemical metabolites of *Fritillaria* thunbergii Miq.——III. Isolation and identification of zhebeinone. Acta Pharm. Sin. (06), 472–475. 1442077

[B67] Zhang Q. J. Zheng Z. F. Yu D. Q. (2011). Steroidal alkaloids from the bulbs of *Fritillaria unibracteata* . J. Asian Nat. Prod. Res. 13 (12), 1098–1103. 10.1080/10286020.2011.619980 22115033

[B68] Zhang Z. He Q. Wu C. Chen X. Zheng Z. (2016). Effects of Chinese herb extracts peiminine on gene expression of HCT-116 cells in patients with colon cancer. J. Traditional Chin. Med. 57 (17), 1504–1509. 10.13288/j.11-2166/r.2016.17.017

[B69] Zhang H. Xu X. Liu Z. Sun-Waterhouse D. Wang J. Ma C. (2021). Effects of edpetiline from *Fritillaria* on inflammation and oxidative stress induced by LPS stimulation in RAW264.7 macrophages. Acta Biochim. Biophys. Sin. (Shanghai) 53 (2), 229–237. 10.1093/abbs/gmaa160 33399208

[B70] Zhang Y. Zhang J. Yan J. Qi X. Wang Y. Zheng Z. (2024). Application of fermented Chinese herbal medicines in food and medicine field: from an antioxidant perspective. Trends Food Sci. and Technol., 148.

[B71] Zhaoli Z. Juan C. Yuzi C. Rihong Z. Hao W. Rui Y. (2023). Antihypertensive activity of different components of Veratrum alkaloids through metabonomic data analysis. Phytomedicine, 120155033. 10.1016/J.PHYMED.2023.155033 37647672

[B72] Zheng Z. He Q. Xu L. Cui W. Bai H. Zhang Z. (2016). The peiminine stimulating autophagy in human colorectal carcinoma cells via AMPK pathway by SQSTM1. Open Life Sci. 11 (01), 358–366. 10.1515/biol-2016-0047

[B73] Zheng Z. Xu L. Zhang S. Li W. Tou F. He Q. (2017). Peiminine inhibits colorectal cancer cell proliferation by inducing apoptosis and autophagy and modulating key metabolic pathways. Oncotarget 8 (29), 47619–47631. 10.18632/oncotarget.17411 28496003 PMC5564592

[B74] Zhou J. L. Xin G. Z. Shi Z. Q. Ren M. T. Qi L. W. Li H. J. (2010). Characterization and identification of steroidal alkaloids of *Fritillaria* species using liquid chromatography coupled with electrospray ionization quadrupole time-of-flight tandem mass spectrometry. J. Chromatogr. A 1217 (45), 7109–7122. 10.1016/j.chroma.2010.09.019 20926090

[B75] Zhou Q. Peng C. Lu T. Xiong L. Liu J. Guo L. (2016). Chemical constituents from *Fritillaria unibracteata* . J. Chin. Med. Mater. 39 (10), 2237–2239.

[B76] Zhu Y. (2010). *Fritillaria* diaphragmatic function and antioxidant protection of an experimental study. Chin. J. Ethnomedicine Ethnopharmacy 19 (11), 32–33.

