# Supplementary material for: Alkaloids from Fritillariae Cirrhosae Bulbus - progress on their pharmacological activities
Source: Front Pharmacol. 2026 May 20;17:1826161. doi: 10.3389/fphar.2026.1826161 (PMC13229978; doi:10.3389/fphar.2026.1826161)
Supplement: Supplementary file 1 [file Supplementaryfile1.pdf]

Literature Search Strategy

Specify the search details:

Databases: PubMed, Web of Science Core Collection, Scopus, CNKI, and WanFang Data.

Search strings (English): (“Fritillariae Cirrhosae Bulbus” OR "*Fritillaria cirrhosa*" OR "Chuanbeimu") AND (“alkaloid” OR "steroidal alkaloid" OR "isosteroidal alkaloid") AND (“pharmacological activity” OR "antitussive" OR "anti-tumor" OR "anti-inflammatory" OR "antibacterial").

Search strings (Chinese): “川贝母” AND “生物碱” AND “药理活性”（或“镇咳”“抗肿瘤”“抗炎”“抗菌”）。

Time range: January 2000–December 2026.

Selection criteria:

Included: Original research articles, reviews, and pharmacopoeial monographs focusing on alkaloids from Fritillariae Cirrhosae Bulbus and their pharmacological effects; studies with clear experimental design and available data on extract characterization.

Excluded: Case reports, conference abstracts, and studies on non-alkaloid metabolites without relevance to the review's focus.
